# Adipose-derived stem cells differentiate to keratocytes in vitro

**Published:** 2010-12-10

**Authors:** Yiqin Du, Danny S. Roh, Martha L. Funderburgh, Mary M. Mann, Kacey G. Marra, J. Peter Rubin, Xuan Li, James L. Funderburgh

**Affiliations:** 1 Department of Ophthalmology, University of Pittsburgh, Pittsburgh, PA; 2 Division of Plastic Surgery, Department of Surgery, University of Pittsburgh, Pittsburgh, PA; 3 McGowan Institute for Regenerative Medicine, University of Pittsburgh, Pittsburgh, PA

## Abstract

**Purpose:**

Adipose-derived stem cells (ADSC) are an abundant population of adult stem cells with the potential to differentiate into several specialized tissue types, including neural and neural crest-derived cells. This study sought to determine if ADSC express keratocyte-specific phenotypic markers when cultured under conditions inducing differentiation of corneal stromal stem cells to keratocytes.

**Methods:**

Human subcutaneous adipose tissue was obtained by lipoaspiration. ADSC were isolated by collagenase digestion and differential centrifugation. Side population cells in ADSC were demonstrated using fluorescence-activated cell sorting after staining with Hoechst 33342. Differentiation to keratocyte phenotype was induced in fibrin gels or as pellet cultures with serum-free or reduced-serum media containing ascorbate. Keratocyte-specific gene expression was characterized using western blotting, quantitative RT–PCR, and immunostaining.

**Results:**

ADSC contained a side population and exhibited differentiation to adipocytes and chondrocytes indicating adult stem-cell potential. Culture of ADSC in fibrin gels or as pellets in reduced-serum medium with ascorbate and insulin induced expression of keratocan, keratan sulfate, and aldehyde dehydrogenase 3 family, member A1 (ALDH3A1), products highly expressed by differentiated keratocytes. Expression of differentiation markers was quantitatively similar to corneal stromal stem cells and occurred in both serum-free and serum containing media.

**Conclusions:**

ADSC cultured under keratocyte-differentiation conditions express corneal-specific matrix components. Expression of these unique keratocyte products suggests that ADSC can adopt a keratocyte phenotype and therefore have potential for use in corneal cell therapy and tissue engineering.

## Introduction

The cornea is the outermost tissue of the eye, providing a protective barrier and a clear path for transmission and refraction of light. This organ comprises three distinct cellular layers: epithelium, stroma, and endothelium. The stroma is primarily responsible for the strength and the refractive properties of the cornea [1]. The optical properties of the stroma result from layers of parallel aligned heterotypic collagen fibrils composed of Types I and V collagen. This collagen is associated with several proteoglycans from the small leucine-rich (SLRP) family, including decorin, lumican, keratocan, and mimecan (osteoglycin) [2-4]. The latter three of these proteins are modified with long, highly sulfated keratan sulfate glycosaminoglycan comprising the corneal keratan sulfate proteoglycans (KSPG), a family of molecules unique to corneal stromal extracellular matrix (ECM). After embryonic development, corneal collagen synthesis decreases, but synthesis of the KSPGs is maintained at a high level, suggesting an essential role for these ECM components in homeostasis of stromal transparency. This role has been confirmed by the loss of transparency associated with reduced sulfated keratan sulfate [5,6] or the loss of keratan sulfate core protein, lumican [7,8]. During stromal wound healing, activated keratocytes transdifferentiate into fibroblasts and myofibroblasts and deposit ECM with reduced corneal KSPGs and a corresponding reduced transparency [9,10]. Similarly, keratocytes in culture rapidly lose KSPG expression [11-14]. The corneal KSPGs, therefore, provide a highly specific marker of keratocyte differentiation that is closely linked with the ability of these cells to maintain corneal transparency.

Corneal blindness affects 8 million individuals worldwide, and currently corneal transplantation is the only method of restoring corneal clarity and function to scarred corneas. This procedure is not without limitations such as immune rejection, graft failure, and limited access to donor-quality tissue. In addition, those countries with the highest rates of corneal blindness often do not have the infrastructure to treat all those burdened with corneal disease [15]. Alternative approaches to transplantation to restore stromal transparency have focused upon corneal tissue engineering [16,17] and more recently stem cell therapy [18,19]. The difficulty in creating a successfully engineered cornea resides in the requirement for a stromal equivalent that retains features and components of the original stroma [18]. Cells to be used for cell therapy and/or tissue engineering would ideally be autologous and with the potential to secrete and organize cornea-specific ECM without eliciting immune rejection.

Adipose-derived stem cells (ADSC) are an abundant population of adult stem cells readily isolated from human adipose tissue. ADSC have multilineage potential and are able to differentiate into fat, bone, cartilage, and muscle under lineage-specific culture conditions [20]. The properties of ease of isolation, high cell yields, immunomodulation, and multipotency qualify the ADSC as an ideal source of autologous stem cells for cell therapy or tissue engineering of corneal stroma.

We recently showed that adult stem cells derived from corneal stroma can be expanded in vitro and can be induced to adopt a keratocyte phenotype, secreting corneal KSPGs and restoring transparency to disrupted corneas [18,21-35]. In this study we investigated the ability of another adult stem cell type, ADSC, to function as corneal stem cells in adopting keratocyte phenotype in vitro. We were able to define culture conditions under which ADSC were induced to express keratocan as well as high molecular weight keratan sulfate.

## Methods

### Cells and materials

Human corneal stromal stem cells (CSSC) and corneal fibroblasts (CF) were isolated and cultured as previously described [21,23]. Briefly, donor human corneas not usable for transplantation were incubated in 1.2 U/ml Dispase II (Roche Diagnostics, Pleasanton, CA) overnight at 4 °C. Epithelial and endothelial cells were removed by dissection and debridement, and the stroma was minced into 2-mm cubes. Stromas were digested up to 3 h at 37 °C in Dulbecco’s modified Eagle’s medium (DMEM) containing 1 mg/ml collagenase type L (Sigma-Aldrich, St. Louis, MO). The resulting primary keratocytes were cultured in a humidified atmosphere containing 5% CO_2_ in DMEM/F-12 (Sigma-Aldrich) with antibiotics [21] for one week before harvesting for RNA. Stem cells from the stromal digest were expanded by culture at a density of 5×10^3^ cells/cm^2^ in a stem cell growth medium (SCGM) consisting of low glucose DMEM:MCDB201 60:40 containing 2% fetal bovine serum (FBS), 10 ng/ml epidermal growth factor (EGF), 10 ng/ml platelet derived growth factor BB (PDGF), 5 ug/ml insulin, 5 ug/ml transferrin, 5 ng/ml selenium, 200 U/ml LIF, and antibiotics/antimycotics [21]. Fibroblastic differentiation was induced by 3 or more passages in DMEM/F-12 with 10% FBS.

Human subcutaneous adipose tissue was obtained from patients undergoing elective lipoaspiration surgery with informed consent under a protocol approved by the Institutional Review Board (IRB) of the University of Pittsburgh, consistent with the principles of the Declaration of Helsinki. Adipose-derived stem cells were isolated by collagenase digestions and differential centrifugation as previously described [24]. Primary adipose-derived cell mixtures were cultured at 5×10^4^ cells/cm^2^ in SCGM. When the cells reached 80% confluency they were passaged 1:4 using trypsin. For flow cytometric analysis trypsizined ADSC were incubated at 1×10^6^ cells/ml in DMEM with 5 µg/ml Hoechst 33342 dye for side population cell sorting [23] and collected for further culture. Adipocyte differentiation medium contained DMEM with 17 µM D-pantothenic acid (Sigma-Aldrich), 0.5 µM dexamethasone (Sigma-Aldrich), 0.2 nM triiodothyronine (Sigma-Aldrich), and 1 µM ciglitazone (Enzo Life Sciences, Plymouth Meeting, PA). Chondrocyte differentiation was induced in DMEM/MCDB201, 2% FBS, 0.1 mM ascorbic acid-2-phosphate, 10^−7^ M dexamethasone, 10 ng/ml recombinant transforming growth factor beta 1 (TGFß1; Sigma-Aldrich) and 100 µg/ml sodium pyruvate. Basal keratocyte differentiation medium (KDM) contained Advanced DMEM (Invitrogen, Rockville, MD) supplemented with 10 ng/ml fibroblast growth factor 2 (FGF2) and 0.1 mM ascorbic acid-2-phosphate (A2P). Heparin-stripped, platelet-poor horse serum (HSHS) [13] was added as noted. Bovine corneal extract in DMEM/F-12 as an alternative to KDM was also used to compare keratocyte gene expression after induction [25]. Antibodies used included anti-keratocan peptide antibody (KeraC) [11] and J19 or J36 monoclonal antibodies to keratan sulfate [23]. Secondary antibodies for western blotting, peroxidase-labeled anti-mouse and anti-rabbit IgG, were from Santa Cruz Biotechnology (Santa Cruz, CA). For fluorescence staining, Alexa Fluor 488 anti-mouse IgG and anti-rabbit IgG and nuclear dye TO-PRO-3 were obtained from Invitrogen.

### Side population cell sorting

ADSC were isolated as a side population on a high-speed cell sorter (MoFlo; DakoCytomation, Fort Collins, CO) using 350 nm excitation and 450nm emission in a method similar to that previously described for corneal stromal stem cells [23]. Verapamil was added to validate side population isolation. After sorting, side population cells were cloned by limiting dilution, maintained in stem cell growth medium (SCGM) and passaged 1:3 by trypsinization when subconfluent.

### Pellet and fibrin gel culture

For pellet culture, 2×10^5^ passage-4 ADSC were collected in a conical bottom 15-ml tube and centrifuged at 400× g for 5 min to form a pellet. The pellets were cultured in SCGM for 3 days, then transferred into various differentiation media which were changed every 3 days for up to 3 weeks. For fibrin gel culture, 33 µl suspension of 12×10^6^ cells/ml passage-4 ADSC were seeded into a fibrin gel consisting of 134 µl of 5 mg/ml human fibrinogen (Sigma-Aldrich) and 33 µl of 100 U/ml bovine thrombin (Sigma-Aldrich). The gel formed in a cell culture incubator (37 °C, 5% CO_2_) for 1 h, and then SCGM containing 1 mg/ml ε-amino-N-caproic acid (Sigma-Aldrich) was added for 3 days. The medium was replaced with KDM containing ε-amino-N-caproic acid at day 3 and changed at 3-day intervals. Human corneal fibroblasts (CF) [18] were used as control for pellet culture and fibrin gel culture. The CF were cultured under the same conditions as ADSC. Media were collected for western blot to detect the expression of keratocan and keratan sulfate after ion exchange isolation of proteoglycans (described below). Cells from the same cultures were lysed to make RNA for RT–PCR or quantitative PCR or were fixed for immunostaining.

### Quantitative RT–PCR (qPCR)

Cell pellets and cells in fibrin gels were stored in a stabilizing reagent (RNAlater; Invitrogen, Austin, TX) for 1 day. RNA was then isolated using the RNeasy mini kit (Qiagen, Valencia, CA) [23], treated with DNase I (Invitrogen) and concentrated by alcohol precipitation. cDNA was transcribed from the RNA using SuperScript II reverse transcriptase (Invitrogen), following recommendations of the manufacturer. qPCR of cDNA was performed using assays containing fluorescent hybridization probes (TaqMan; Applied Biosystems, Foster City, CA) or with direct dye binding (SYBR Green; Applied Biosystems) as previously described [21]. Primers for SYBR assays were designed using online software (Primer 3) with the sequences shown in Table 1. Amplification of 18S rRNA was performed for each cDNA (in triplicate) for normalization of RNA content. A negative control lacking cDNA was also included in each assay. Relative mRNA abundance was calculated as the Ct for amplification of a gene-specific cDNA minus the average Ct for 18S expressed as a power of 2 (2^–ΔΔCt^). Three individual gene-specific values thus calculated were averaged to obtain mean±SD.

**Table 1:** RT–PCR primers.

**Gene Name**	**Gene ID**	**Primer sequence**
Leptin	NM_000230	Forward: TCCTGGATTCCTTTCCTTCA
		Reverse: CAATCGAGGAGGGCAGAATA
Keratocan	NM_007035	Forward: ATCTGCAGCACCTTCACCTT
		Reverse: CATTGGAATTGGTGGTTTGA
*ALDH3A1*	NM_001135168	Forward: CATTGGCACCTGGAACTACC
		Reverse: GGCTTGAGGACCACTGAGTT
18S Ribosomal RNA	NR_003286	Forward: CCCTGTAATTGGAATGAGTCCAC
		Reverse: GCTGGAATTACCGCGGCT

### Western blotting

Proteoglycans were recovered from culture media by ion exchange chromatography on microcolumns (SPEC-NH_2_; Agilent Technologies, Wilmington, DE), as described previously [23]. Proteoglycans were digested with a mixture of keratanase II and endo-β-galactosidase [13]. Digested and undigested samples were run on a 4%–20% SDS–PAGE gel, transferred to polyvinylidene difluoride (PVDF) membrane and subjected to immunoblotting with KeraC antibody against keratocan [13] and antibody J36 against keratan sulfate [21].

### Histology

Monolayer cells were rinsed briefly in phosphate-buffered saline (PBS), fixed for 12–15 min in 3% paraformaldehyde in PBS at room temperature, and rinsed in PBS. Oil red O (Sigma-Aldrich) was prepared at 0.5% in isopropanol, diluted to 0.3% in water and filtered before use. Cells were stained with oil red O for 15 min and rinsed with 60% isopropanol followed by hematoxylin stain for nuclei. Bright-field micrography was performed with a 40× oil objective. Pellets and fibrin gels were rinsed briefly in PBS, fixed for 15 min in 3% PFA in PBS at room temperature, rinsed in PBS, embedded in optimal cutting temperature embedding compound (Tissue-Tek OCT; Electron Microscopy Sciences, Hatfield, PA), frozen, and stored at −20 °C until they were cut as 8-µm sections on a cryostat. Sections were hydrated in PBS before staining. Nonspecific binding was blocked with 10% heat-inactivated goat serum. Sections were incubated for 1 h at room temperature with primary antibodies. After two rinses in PBS, secondary antibodies and nuclear counterstain (TO-PRO-3; Invitrogen) were added for 1 h at room temperature. The samples were photographed using a confocal microscope with a 20× oil objective (Bio-Rad Laboratories, Hercules, CA).

## Results

### Side population cells in ADSC

**Figure 1:**
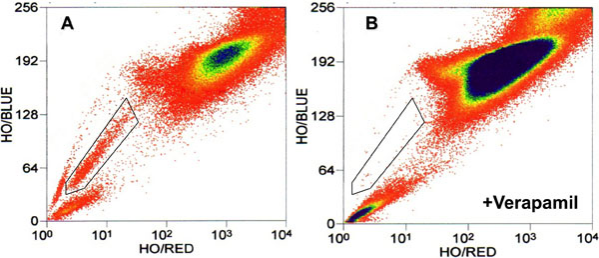
Flow cytometric identification of side population from cultured human adipose-derived stem cells (ADSC). **A**: Passage-two ADSC stained with Hoechst 33342 were analyzed using 350-nm excitation with blue (635 nm) and red (488 nm) emission. Cells showing reduction of both blue and red fluorescence (side population cells) were analyzed as defined by the box outlined on the left. **B**: An analysis similar to (A) but with a preincubation in 50 μM verapamil before incubation with Hoechst 33342.

Human ADSC isolated by collagenase and differential centrifugation were labeled with Hoechst 33342 dye and analyzed using flow cytometry to identify side population cells (Figure 1). This technique originally described by Goodell et al. [26] identifies cells that efflux the Hoechst 33342 dye as a result of the expression of ATP-binding cassette (ABC) transporter proteins. Side population cells are present in small numbers in many tissues and have been found to exhibit adult stem cell-like properties [27,28]. Figure 1A demonstrates that a population (defined by the box) amounting to less than 1% of the total cells shows reduced Hoechst 33342 dye staining and a color shift toward blue in the cultured ADSC. When we preincubated with verapamil, an inhibitor of ABC transporter function, the population was eliminated (Figure 1B). The presence of this characteristic side population suggests the presence of multipotent adult stem cells in the ADSC [27,28].

### Differentiation potential of ADSC

**Figure 2:**
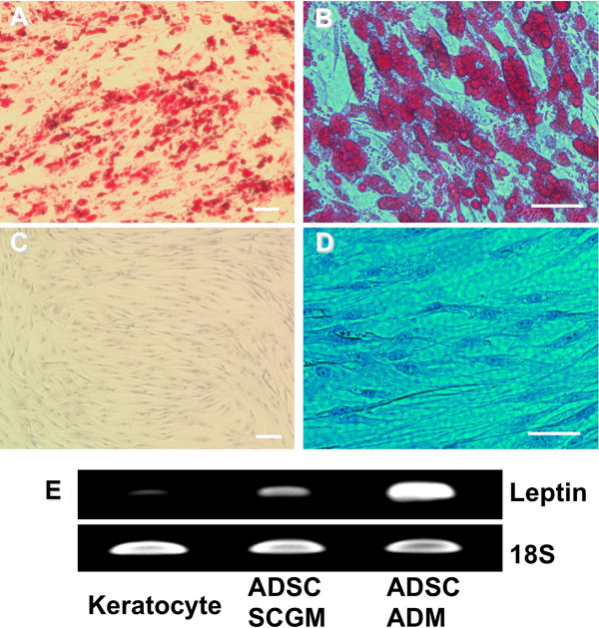
Induction of adipocytes from ADSC. ADSC were induced to differentiate into adipocytes in ADM for two weeks as described in Methods. **A**, **B**: ADSC were fixed and stained with Oil Red O. **C**, **D**: ADSC without induction were stained with Oil Red O as control. **E**: mRNA for leptin was detected by RT–PCR. Leptin expression (Upper) and 18S (Lower). Lane 1, uncultured keratocytes; lane 2, ADSC; lane 3, ADSC in adipocyte induction medium. Scale bars: 100 μm (**A**, **C**); 50 μm (**B**, **D**).

To demonstrate multipotent differentiation potential, ADSC were expanded and grown in under conditions that induce differentiation to mature adipocytes in adult stem cells. Figure 2A,B shows staining with oil red O, which stains the characteristic neutral triglycerides and lipids in adipocytes. Well defined lipid droplets were present within differentiated cells. Figure 2C,D shows the lack of oil red O staining in ADSC cultured in SCGM. Expression of the leptin gene encoding an adipokine secreted by mature adipocytes [29] was increased in ADSC after culture in ADM (Figure 2C). Leptin was minimally expressed by keratocytes, but was upregulated in multipotent stromal corneal stem cells as well as in ADSC.

**Figure 3:**
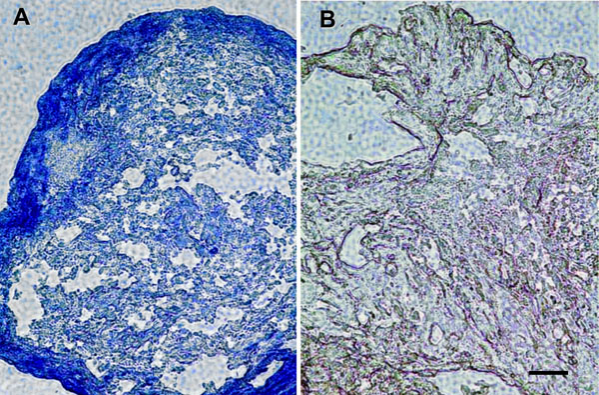
Induction of cartilage matrix expression by ADSC. ADSC (**A**) and CF (**B**) were cultured as pellets (2×10^5^) in chondrocyte differentiation medium for three weeks. The pellets were fixed, imbedded in OCT, cut into 8 µm thick sections and stained with toluidine blue to detect proteoglycan staining typical of cartilage. Scale bar indicates 50 μm.

ADSC were also cultured under conditions reported to induce chondrogenic differentiation. After 3 weeks in chondrocyte differentiation medium, as shown in Figure 3A, ADSC secreted cartilage matrix as indicated by positive toluidine blue staining for proteoglycans characteristic of cartilage [30]. In contrast, CF grown in pellet culture did not display chondrogenic differentiation as evidenced by absence of toluidine blue staining (Figure 3B).

### Expression of keratocyte markers

**Figure 4:**
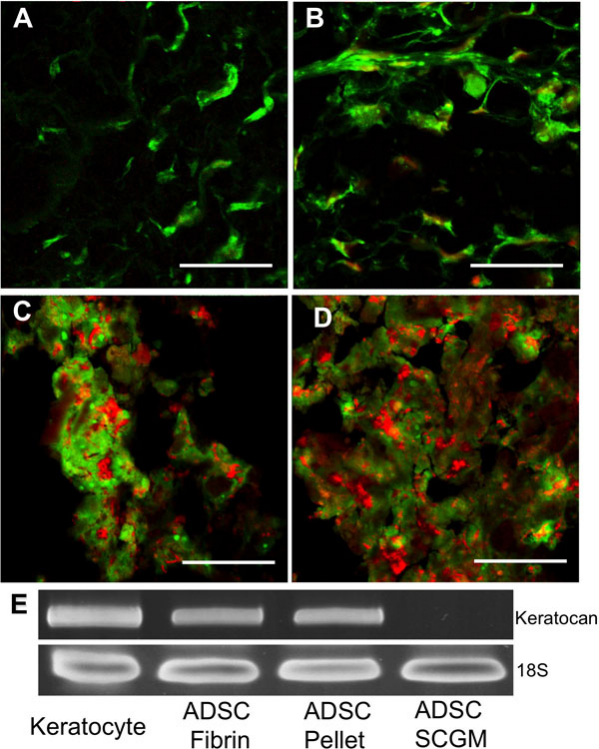
Induction of keratocyte markers in ADSC. **A**, **B**: ADSC were cultured in fibrin gels for 3 weeks in keratocyte differentiation medium. **C**, **D**: ADSC were cultured as pellet for 3 weeks in keratocyte differentiation medium. Immunofluorescent staining shows the presence of keratan sulfate with antibody J19 (green; **A**, **C**) or keratocan with antibody KeraC, (green; **B**, **D**). Red shows nuclear staining by propidium iodide. **E**: RT–PCR shows keratocan expression in (left to right) uncultured keratocytes (positive control), ADSC in fibrin gel, ADSC as pellet culture, ADSC in SCGM. Scale bars=20 μm.

Given the clear multipotent nature of ADSC, we investigated the ability of these cells to assume a keratocyte phenotype using methods previously successful in differentiating human corneal stromal stem cells into keratocytes [21]. ADSC were seeded in fibrin gels (Figure 4A,B) and cultured as pellets (Figure 4C,D) and transferred into KDM for 3 weeks. Immmunostaining of ADSC cultures the showed presence of the stroma-specific ECM molecules keratocan and keratan sulfate in both the fibrin gels and pellets (Figure 4). ADSC in fibrin gels were more sparsely distributed (Figure 4A,B) than in the pellet cultures (Figure 4C,D). Expression of keratocan in ADSC incubated in KDM was confirmed with RT–PCR using human keratocan primers (Figure 4E). ADSC maintained in SCGM alone did not express keratocan mRNA. In addition, we observed (not shown) that ADSC in KDM formed extensive cell-cell contacts similar to those connecting keratocytes. Cultures using both fibrin gel and pellet methods induced ADSC differentiation into keratocyte-like cells.

**Figure 5:**
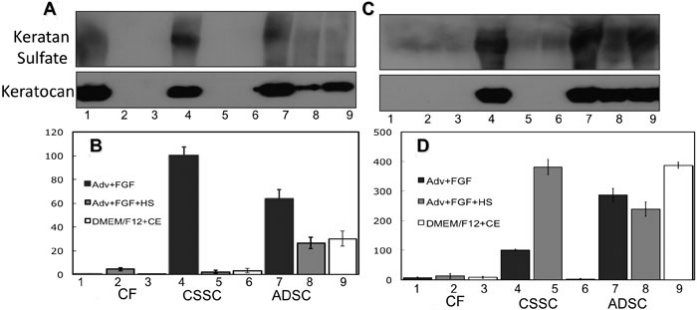
Keratan sulfate, keratocan protein, and mRNA expression by CSSC, ADSC, and CF in different media. **A**, **B**: Fibrin gel cultures after 3 weeks. **C**, **D**: Pellet cultures after 3 weeks. **A** and **C** are western blots showing keratan sulfate and keratocan. **B** and **D** show qPCR data of keratocan mRNA. Samples 1–3: CF, 4–6: CSSC cells; 7–9, ADSC cells. Samples 1, 4, 7: keratocyte differentiation medium; Samples 2, 5, 8: keratocyte differentiation medium + 1% HSHS. Samples 3, 6, 9: DMEM/F-12 medium with bovine corneal extract (1:10) [25]. Expression of mRNA is shown normalized to monolayer of CSSC in keratocyte differentiation medium=100.

After we established the differentiation potential of ADSC into cells synthesizing keratocyte-specific proteins, we examined the effects of varying culture conditions on both keratocan and keratan sulfate expression levels. Using a combination of three different culture media and fibrin gel or pellet culture, we found that ADSC in pellet cultures (Figure 5C,D) had more consistent expression of both keratocan and keratan sulfate at the protein and mRNA level than ADSC in fibrin gels (Figure 5A,B). This was similar to human CSSC, which have elevated keratocan expression in pellet cultures compared to fibrin gel culture (Figure 5A,B) [21]. In fact, the level of keratocan mRNA in ADSC in pellet culture was similar to that of stem cells from corneal stroma (CSSC; Figure 5D). Bovine corneal extract [25] appeared to enhance differentiation of the ADSC but had little effect on CSSC (Figure 5, samples 6 and 9).

**Figure 6:**
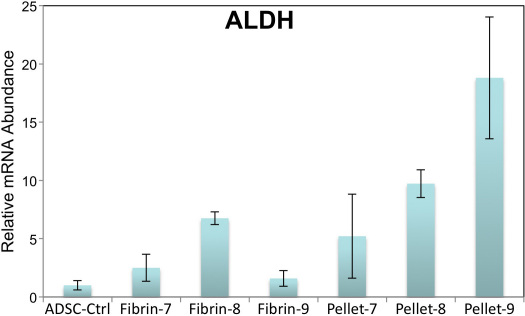
ALDH is upregulated in ADSC cultured under conditions that induce keratocyte differentiation. Expression of ALDH3A1 was compared in ADSC cells cultured as described in Figure 5 using qPCR as described in Methods. Expression levels were normalized to that of ADSC in SCGM (ADSC-Ctrl) in samples in Fibrin or Pellets under conditions 7, 8, 9 as described in Figure 5.

The gene ALDH3A1 codes for the widely distributed protein aldehyde dehydrogenase (ALDH).  In differentiated cells of the cornea, however, ALDH is exceptionally abundant, especially in keratocytes, where it makes up as much as 40 % of soluble protein [31]. Previously we observed ALDH to be markedly upregulated as CSSC differentiate to keratocytes [21] thus, we would expect ALDH upregulation if ADSC are adopting keratocyte phenotype. In Figure 6 we documented strong upregulation of this corneal marker mRNA, particularly in the pellet cultures of ADSC.

**Figure 7:**
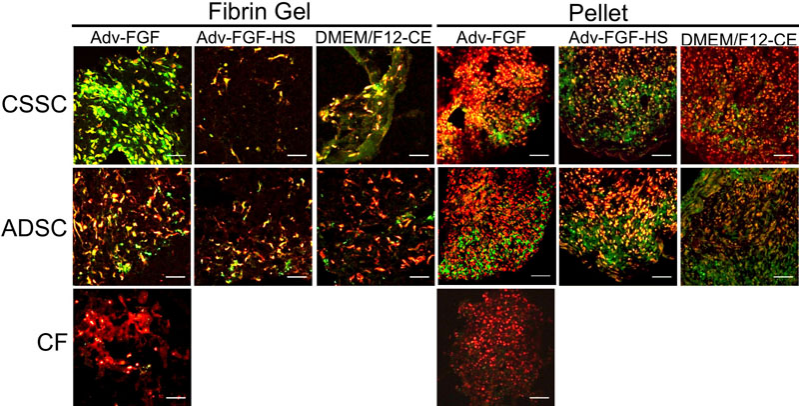
Keratan sulfate staining in CSSC, ADSC, and CF cultured in fibrin gels or as pellets in different media. Frozen sections of pellet and fibrin gel cultures were stained with antibody J19 against keratan sulfate (green) and nuclei (red) after 3 weeks of culture in different media. Abbreviations: CSSC: corneal stromal stem cells, ADSC: Adipose-derived stem cell, Adv: Advanced DMEM, FGF: fibroblast growth factor 2, HS: heparin stripped horse serum, CE: bovine corneal extract. Scale bars=50 μm.

**Figure 8:**
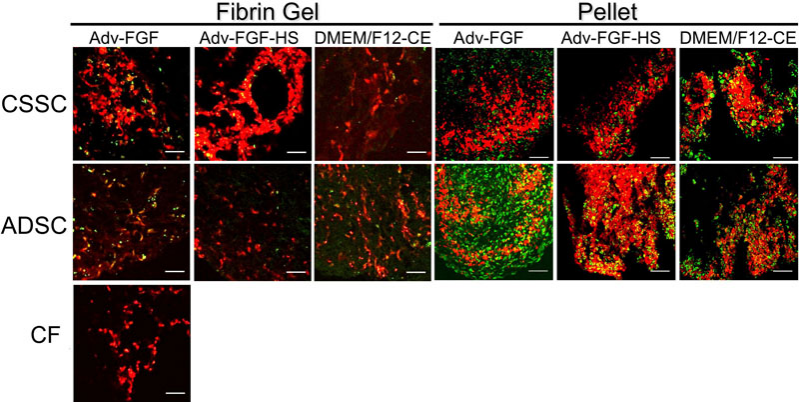
Keratocan staining in CSSC, ADSC, and CFs cultured in different media. Frozen sections of pellet and fibrin gel cultures were stained with antibody KeraC for keratocan (green) and cell nuclei (red) after 3 weeks of culture in several different media. Abbreviations: CSSC: corneal stromal stem cells, ADSC: Adipose-derived stem cell, Adv: Advanced DMEM, FGF: fibroblast growth factor 2, HS: heparin stripped horse serum, CE: bovine corneal extract. Scale bar=50 μm.

Immunostaining of keratan sulfate (Figure 7) and keratocan (Figure 8) demonstrated that the mRNA increases documented in Figure 5 correlate with accumulation of these keratocyte-specific markers in the ECM of the cultures. Consistent with mRNA levels, accumulation of these matrix molecules was more evident in pellet cultures, and CSSC and ADSC generated similar amounts. This result was in contrast to CF which did not consistently synthesize keratocan or keratan sulfate when grown in either fibrin gels or pellets.

## Discussion

In this study we have shown that ADSC isolated from lipoasiprate have the potential to differentiate in vitro into cells that synthesize and secrete keratocyte-specific proteins as confirmed by both immunohistochemical and molecular evidence. Throughout our study we used ADSC grown and expanded at clonal density. These cells were cultured in various differentiation conditions while maintaining their ability to differentiate into adipocyte (Figure 2) and chondrocyte (Figure 3) lineages similar to results reported previously [32]. In addition, we show that ADSC grown in three-dimensional fibrin gel and pellet culture systems supplemented with appropriate differentiation medium can be induced to differentiate into a keratocyte lineage (Figure 4, Figure 5, and Figure 6). This differentiation is evidenced by the high levels of cornea-specific keratocan mRNA and protein expression and the increased presence of keratan sulfate in the culture medium. Expression of aldehyde dehydrogenase 3 family, member A1 (ALDH3A1), keratocan, and keratan sulfate by ADSC was observed in several different media and in both culture formats.

Our previous work showed that keratan sulfate is 10^3^- to 10^6^ fold more enriched in cornea than any other tissue [33] and that synthesis of keratan sulfate by keratocytes is highly regulated in vitro and in vivo [14,34,35]. Keratan sulfate biosynthesis in vitro, therefore, represents the most stringent marker of the keratocyte phenotype yet described. The observation that ADSC can be induced to produce keratan sulfate is novel and presents the best evidence to date that these cells can adopt the keratocytes phenotype. Keratocan is highly enriched in keratocytes, as is ALDH3A1. Neither of these proteins represents a unique corneal marker but like keratan sulfate, both, are highly expressed in keratocytes and upregulated as CSSC differentiate to keratocytes [21]. Upregulation of keratocan and *ALDH3A1* mRNA simultaneously with synthesis of keratan sulfate by ASSC strengthens the argument that these cells are indeed differentiating to keratocytes.

High cell density, as occurs in pellet cultures, rather than dendritic cell morphology, appeared to positively influence keratocyte differentiation potential. The pellet cultures produced a denser, more abundant ECM with higher keratocan and keratan sulfate. This is similar to what we observed in human CSSC, which differentiate and express higher levels of keratocan and keratan sulfate in pellet cultures [21,36]. Compared to fibrin gels, ADSC expressed higher keratocan and keratan sulfate in pellet cultures (Figure 5). Although pellet cultures clearly influenced keratocan expression, the addition of extra supplementary factors such as horse serum and bovine extract did not appear to significantly enhance levels of keratocan protein. Thus, keratocyte differentiation of ADSC appears to be more dependent on the three-dimensional culture environment and less dependent on exogenous molecular supplementation. Growth of differentiated keratocytes based on the architecture of the culture conditions, rather than a complicated menu of biologically active molecules, may be advantageous to the future isolation and production of clinically useful cells while lowering the risk of contaminants from additions.

ADSC are an abundant and readily accessible source of multipotent adult stem cells with the desirable potential for autologous cell therapy, thereby presenting the potential for personal tissue engineering of the corneal stroma. A recent study by Arnalich-Montiel et al. [19] demonstrated that human lipoaspirate-derived cells could be transplanted into the corneal stroma of rabbits. Under these conditions, the ADSC did not elicit a significant immune response, remained viable, and could be immunostained for ALDH and keratocan [19]. The current study builds on these findings, using the more stringent keratocyte phenotypic marker keratan sulfate and defining in vitro conditions under which the keratocyte phenotype is expressed by these cells. Understanding these conditions will allow development of use of ADSC in stromal bioengineering applications.

The immunomodulatory effects of ADSC are another important aspect of their potential use in cell based therapy. These effects have been attributed to a lack of HLA-DR expression and active suppression of the proliferative T-cell response [37]. ADSC have been shown to enhance dermal wound healing by the secretion of a variety of soluble growth factors accelerating wound repair and regeneration [38]. In addition, ADSC were shown to increase wound healing through differentiation into cell types capable of replacing and regenerating damaged tissue [39]. The biologic effect these soluble factors have in corneal wounds remains to be determined, but could include production of the correct ECM and maintenance of a keratocyte phenotype. Particularly relevant to the environmental exposures of the cornea, ADSC have been shown to provide protective antioxidant effects against chemically- and UVB-induced reactive oxygen species [40,41].

Athough KSPGs and ALDH are markers of the keratocyte phenotype, the true test of keratocyte function is elaboration of the highly organized, transparent ECM of the corneal stroma. Thus, the potential for ADSC in corneal cell therapy and tissue engineering will require further examination before clinical application. Our previous work identifying the molecular markers for keratocytes [21,23] will allow us to determine how far along the differentiation pathway the ADSC have traveled. It will be important to determine the long-term fate of ADSC-derived keratocyte-like cells and the surrounding stromal cells. ADSC have the potential to secrete many molecules that may influence wound healing in a manner detrimental to corneal transparency.

In conclusion, our results provide novel evidence documenting the potential of ADSC to adopt a keratocyte phenotype in vitro. These results confirm and extend the results of an earlier in vivo study [19]. Although more detailed molecular characterization of the tissue elaborated by the ADSC will be necessary, the demonstration that non-ocular adult stem cells have potential to generate corneal ECM in vitro opens an important potential for bioengineering of corneal tissue using autologous cells.
